# Influence of Social Crowding on Rumor Refutation: The Mediating Effect of Impression Management and Social Connectedness

**DOI:** 10.3390/bs16050803

**Published:** 2026-05-18

**Authors:** Zhaoyang Sun, Mengchan Yuan, Haolin Xuan, Wan Ni, Li Zhang

**Affiliations:** 1 School of Journalism and Communication, Shandong University, Jinan 250100, China; zhaoysun@sdu.edu.cn; 2 Institute of Psychology, Chinese Academy of Sciences, Beijing 100101, China; yuanmc@psych.ac.cn; 3 Department of Psychology, University of Chinese Academy of Sciences, Beijing 100049, China; 4 School of Management, Shandong University, Jinan 250100, China; 202400272139@mail.sdu.edu.cn

**Keywords:** social crowding, willingness to refute rumors, impression management, social connectedness, self-construal

## Abstract

Internet rumor refutation represents a critical issue in the current governance of the Internet information environment. Different from the mainstream research that focuses on refutation subjects, methods, and information presentation formats, this study adopts a psychological perspective at the individual level to examine how a typical environmental factor—social crowding (the subjective psychological experience arising when spatial demand exceeds supply due to high population density per unit area) affects individuals’ willingness to refute rumors, as well as the mediating mechanisms and boundary conditions of this effect. The findings provide implications for motivating individual participation in Internet rumor refutation. Considering rumor refutation as a prosocial behavior, this study integrates the moral judgment framework and focuses on the positive side of greater self-other overlap induced by social crowding. Through one questionnaire survey and two experimental studies, most of the hypotheses are supported. The results indicate that social crowding positively influences willingness to refute rumors, with impression management and social connectedness serving as parallel mediators in this relationship. Additionally, interdependent self-construal positively moderates the relationship between social crowding and social connectedness, whereas the moderating role of independent self-construal was not supported. This study expands online rumor-refutation research from the perspective of environmental antecedents, proposes an altruistic-egoistic dual-pathway model, and provides practical implications for governments and social media platforms in rumor governance.

## 1. Introduction

With the rapid advancement of information dissemination technologies and the increasing influence of social media, the internet has become a primary source of information and a platform for the public to exchange views. However, due to the low barriers to online expression and the inherent difficulties in supervision and regulation, the authenticity and accuracy of online information are often difficult to guarantee. The uneven quality of online information has led to the widespread problem of Internet rumors. Internet rumors refer to unverified information generated or disseminated on social media through specific channels (Zhao et al., 2016). The rapid development of artificial intelligence has also driven Internet rumors toward an intelligent trend, facilitating the customized generation and precise dissemination of rumors. If not promptly clarified, Internet rumors may pose far-reaching consequences: they mislead individual cognition, trigger anxiety, induce group panic, and weaken government credibility. Therefore, combating Internet rumors has become a critical issue in information governance (Lazer et al., 2018).

However, previous studies have primarily focused on the structural features of rumor refutation, noting, for instance, that the velocity and reach of refutation information often lag significantly behind the rumors themselves (Vosoughi et al., 2018), or on macro-level strategies such as institutional interventions by governments and media (Paek & Hove, 2019; Pennycook & Rand, 2019). Such perspectives often treat individuals as passive recipients of corrected information. While recent scholarship has begun to recognize individual agency, noting that user-generated health refutations can be more psychologically resonant and effective than official ones (Kim et al., 2019), there remains a significant gap in understanding the intrinsic psychological motivations that drive ordinary individuals to voluntarily disseminate refutation information. In particular, the situational and environmental antecedents that foster this willingness remain underexplored. Therefore, identifying situational factors that can naturally activate individuals’ intrinsic psychological motivations is a crucial step toward understanding rumor refutation from a bottom-up, individual psychological perspective. Moreover, as a social behavior, rumor refutation unfolds within densely populated social networks and involves the collective participation of numerous individuals. Consequently, social crowding emerges as a particularly noteworthy environmental variable. Given that mobile digital devices now enable individuals to engage in online behavior while immersed in crowded physical settings (Humphreys, 2013), the boundaries between physical and digital environments have become increasingly permeable (de Souza e Silva, 2006). This “hybrid space” allows psychological states induced by physical crowding to spill over into the digital realm, potentially serving as a situational catalyst for subsequent online actions. Therefore, it is worth examining whether social crowding can extend its influence to the digital public sphere, specifically in the context of rumor refutation.

Social crowding refers to the subjective psychological experience that arises when an individual’s need for space exceeds the available supply due to a high density of people within a given area (Shen et al., 2019; Stokols, 1972). It is a pervasive phenomenon in daily life, encountered in settings ranging from subway stations and buses during morning rush hours to supermarkets during sales promotions. Consequently, increasing research attention has been directed toward individuals’ psychological responses and decision-making in social crowding contexts. Classical environmental psychology has long emphasized the deleterious effects of crowding particularly within consumption contexts (Harrell et al., 1980). Being in a crowded environment leads to a sense of frustration from losing control (Hui & Bateson, 1990), which in turn makes them more likely to choose unhealthy foods (Hock & Bagchi, 2018), and lowers their evaluations of products in that setting (O’Guinn et al., 2015). An emerging line of inquiry suggests that crowding can also carry positive implications. For example, social crowding has been found to enhance consumers’ store loyalty (Ha & Lee, 2016), and to promote a slower life history strategy, including greater investment in education (Sng et al., 2017). These positive aspects, however, remain comparatively understudied, and their relevance beyond the marketplace is poorly understood. Building on the premise of boundary permeability between physical and digital spaces (Humphreys, 2013), it is worth examining whether social crowding can extend its influence to the digital public sphere. To address this question, this paper focuses on the influence of social crowding on individuals’ willingness to refute rumors, as well as the underlying mediating mechanisms and boundary conditions.

This paper proposes that social crowding can act as a situational catalyst that enhances individuals’ willingness to refute rumors. Central to this relationship is the concept of self-other overlap, a psychological state where the boundaries between the self and others become blurred due to high physical density (Tan et al., 2015). Adopting this perspective, this study moves beyond the traditional view of crowding as a mere stressor and instead explores how it activates dual motivational pathways—egoistic and altruistic—to drive individuals’ willingness to refute rumors. We propose that impression management and social connectedness play parallel mediating roles in this process.

On the one hand, social crowding-induced self-other overlap threatens the clarity of individuals’ self-concept by blurring the boundary between the self and others (Tan et al., 2015), which activates their self-presentation motivation, and prompts individuals to engage in impression management as a means to re-establish their distinct value (Xu et al., 2012). On the other hand, a closer psychological distance enhances interpersonal intimacy and strengthens perceived social connectedness (Aron et al., 1992). As a behavior that consumes time and cognitive resources while benefiting others and society, Internet rumor refutation can be conceptualized as a prosocial behavior (Tang et al., 2022). Drawing on the moral judgment framework widely employed in prosocial behavior research, both the egoistic motivation of shaping one’s personal image (Brown & Smart, 1991) and the altruistic motivation of providing informational support to others (He & Wei, 2009) constitute utilitarian motives underlying moral decision-making. Therefore, we propose that the enhanced impression management motivation and the social connectedness triggered by social crowding can each independently drive individuals’ willingness to refute rumors. The former is by serving the egoistic desire to present a positive social image, and the latter by fulfilling the altruistic impulse to provide informational support to others. Accordingly, this study examines whether impression management and social connectedness mediate the relationship between social crowding and rumor refutation willingness. Furthermore, to identify the boundary conditions of these pathways, this study adopts a self-other relationship perspective and incorporates two individual cognitive variables—independent self-construal and interdependent self-construal—as potential moderators.

This study develops a theoretical model of how social crowding influences individuals’ willingness to refute rumors. Theoretically, this research expands the behavioral motivation framework in rumor refutation research while also enriching the literature on the positive effects of social crowding. On a practical level, the findings of this study hold significant implications for encouraging individuals to more actively participate in the dissemination of rumor refutation information on online platforms, thereby enhancing the effectiveness of Internet rumor governance and contributing to a healthier online information environment.

## 2. Literature Review

### 2.1. Rumor Refutation: From the Perspective of the Moral Judgment Analysis Framework

Internet rumors refer to unverified information generated or disseminated on social media through specific channels (Zhao et al., 2016). Their widespread diffusion can trigger a series of negative consequences including information distortion and public panic (Chao et al., 2025; Sarraf et al., 2024). Therefore, timely and effective Internet rumor refutation is of critical importance. Internet rumor refutation refers to the release of authoritative and scientific clarifications targeting rumor content after its spread, counteracting the dissemination of rumor-related information in the online space and thereby mitigating the negative impacts of false rumors (Paek & Hove, 2019). Zhao et al. (2016) found that expectations of harmful consequences of rumor dissemination can prompt individuals to engage in rumor refutation. Tang et al. (2022) noted that personal norms, altruism, social relationships, and knowledge influence individuals’ behaviors against rumors.

As a voluntary, time- and effort-consuming behavior that helps others identify information risks, make appropriate decisions (Wang et al., 2022), and benefit others and society, rumor refutation can be regarded as an online prosocial behavior (De Groot & Steg, 2010; Tang et al., 2022). However, few studies have examined it from the perspective of prosocial behavior. The moral judgment framework is a typical theoretical perspective in prosocial behavior research, which analyzes the various motivations underlying individuals’ moral and utilitarian decision-making. Accordingly, this paper takes the moral judgment framework as the theoretical foundation to analyze the psychological motivations underlying individuals’ active participation in Internet rumor refutation.

The moral judgment framework distinguishes between two types of motivations for prosocial behavior: deontological motivation and utilitarian motivation. Deontological motivation stems from internalized moral norms (De Groot & Steg, 2010). In contrast, utilitarian motivation involves goal-oriented, rational trade-offs aimed at maximizing benefits, which can be further divided into altruistic public interests and egoistic self-interests. According to the dual-process framework of moral judgment, deontological decision-making is relatively fast and automatic, and individuals’ internal moral values are difficult to modify in a short time. Utilitarian decision-making, however, is relatively slow and controllable, such that individuals’ egoistic and altruistic motives are more susceptible to external environmental influences.

Previous studies on Internet rumor refutation have mostly focused on deontological motivation, namely, individuals’ moral norms (Tang et al., 2022; Zhao et al., 2016). Nevertheless, rumor refutation occurs in social media environments, where all behaviors are inherently embedded in interactions between individuals. Therefore, this paper proposes that individuals’ motivation to engage in Internet rumor refutation may also derive from utilitarian pursuits, which can be further categorized into altruistic and egoistic motives. Specifically, individuals’ motivation to refute rumors may include the pursuit of public interests: when individuals forward rumor-refuting information, they satisfy the informational needs of recipients, correct others’ cognitive biases, and alleviate anxiety stemming from information deficits or uncertainty, thereby exerting positive effects on the broader online space and society as a whole. In this case, the utilitarian motivation is altruistic. On the other hand, individuals’ motivation may also involve the pursuit of self-interests: by engaging in Internet rumor refutation, individuals can present themselves in online space, construct a positive self-image as caring, kind, and rational, and enhance their reputation. In this case, the utilitarian motivation is egoistic.

### 2.2. Social Crowding

Social crowding refers to the subjective psychological experience that occurs when the number of people per unit area in an individual’s surrounding environment leads to insufficient space to meet the individual’s needs, resulting in feelings of constraint, restriction, and confinement experienced by the individual in high-density populations (Li et al., 2009; Shen et al., 2019; Stokols, 1972). Early research treated crowding simply as physical density (Hall, 1966; McGrew, 1970), but subsequent scholars have pointed out that social crowding places greater emphasis on the subjective psychological experience triggered by high population density within a specific physical environment (Desor, 1972; Stokols, 1972).

Social crowding exerts extensive impacts on individuals’ psychology and behaviors across multiple dimensions. Classical environmental psychology, often grounded in stimulus overload theory, has predominantly documented the deleterious effects of crowding. Its most direct consequence is the invasion of individuals’ personal space (Maeng et al., 2013), which further triggers a sense of threat (Worchel & Teddlie, 1976) and reduces their freedom of movement and perceived control over the surrounding environment (Consiglio et al., 2018; Hock & Bagchi, 2018; Stokols, 1972; van Rompay et al., 2008). Under such stress, individuals tend to exhibit avoidance or withdrawal behaviors: they become more risk-averse (Machleit et al., 1994), prefer familiar brands, reduce exploratory behaviors in stores (Harrell et al., 1980), and decrease interactions with others in the shopping environment as well as their duration of stay (Huang et al., 2018). Accordingly, when individuals perceive their behavior as constrained, they tend to exhibit psychological and behavioral withdrawal (Hui & Bateson, 1990).

However, this “negative-only” view may be incomplete. Emerging lines of inquiry suggest that crowding can also carry positive interpersonal implications. Individuals may adopt strategies to restore their sense of control, such as strengthening connection and attachment with brands (Huang et al., 2018), and engaging in prosocial behaviors including word-of-mouth communication (Consiglio et al., 2018). In terms of social interaction, social crowding facilitates communication among individuals by reducing social tension and prejudice through frequent contact (Allport, 1954), further alleviates negative emotions such as anxiety, promotes tolerance and acceptance of others, and helps establish emotional bonds with others (Eroglu et al., 2005), thereby enhancing their sense of social connectedness (Stieler & Germelmann, 2016). Beyond the marketplace, social crowding has been found to promote a slower life history strategy, including greater investment in education (Sng et al., 2017). These findings collectively suggest that social crowding, rather than being a purely aversive stressor, possesses the potential to function as a positive situational driver.

Crucially, in the era of mobile internet, the psychological impact of social crowding is no longer confined to the immediate physical setting. As individuals increasingly engage with mobile devices while immersed in high-density environments, the boundaries between physical and digital spaces have become permeable (de Souza e Silva, 2006; Humphreys, 2013). This “hybrid space” allows the psychological states induced by physical density to logically spill over into the digital realm. Therefore, this study proposes that the positive effects of social crowding may also extend to online rumor refutation. Specifically, social crowding simultaneously activates individuals’ egoistic and altruistic motivations, making them more inclined to refute rumors in crowded environments. Correspondingly, this study puts forward two parallel mediating pathways: impression management and social connectedness.

**H1.**  *Social crowding has a significant positive impact on individuals’ willingness to refute rumors.*

### 2.3. The Mediating Role of Impression Management

First, social crowding enhances individuals’ motivation for impression management. Impression management refers to the process by which individuals influence others’ perceptions of themselves through certain means (Baumeister, 1982). The prevalence of social media has extended impression management to the online domain, enabling individuals to shape their personal image through internet platforms. In social crowding environments, the psychological distance individuals perceive from others decreases with spatial distance (Zhang & Wang, 2009), leading to increased self-other overlap (Tan et al., 2015), which in turn blurs self-boundaries and reduces self-concept clarity (Campbell, 1990). In response, to maintain an independent sense of self, individuals instinctively seek to differentiate themselves from others (Markus & Kitayama, 1991), thereby expressing their existence through behaviors and choices—such as a greater preference for unique products (Xu et al., 2012) and the use of self-expressive items like clothing to highlight personal identity (Morrison & Johnson, 2011). Additionally, the “spotlight effect” triggered by social crowding causes individuals to overestimate others’ attention to themselves, thereby enhancing self-monitoring and concerns about negative evaluations (Gilovich et al., 2000), which further strengthens their motivation for impression management. In summary, individuals in social crowding environments have a stronger desire to convey their views and present their self-image, and thus exhibit higher impression management motivation.

Motivation for Impression management further increases individuals’ willingness to refute rumors. Previous research has shown that impression management motivation is a key driver of behaviors related to moral responsibility (Sripada, 2016). When individuals’ impression management motivation increases, they engage more in prosocial behaviors to shape their self-image (Brown & Smart, 1991). As a form of prosocial behavior, rumor refutation possesses attributes of self-expression and impression management. Compared to offline interventions, online rumor refutation offers a socially visible yet physically safe platform for individuals to demonstrate influence, acquire a prosocial reputation, and present a positive self-image to others. Therefore, from an egoistic perspective, individuals participate in rumor refutation to shape their self-image, signal their willingness and ability to engage in prosocial behavior, and make others perceive them as just and virtuous, thereby achieving a prosocial reputation in the online public sphere. Accordingly, the following hypothesis is proposed:

**H2.**  *Impression management mediates the relationship between social crowding and individuals’ willingness to refute rumors.*

### 2.4. The Mediating Role of Social Connectedness

Social crowding also enhances individuals’ perceived social connectedness. Lee and Robbins (1995) first defined social connectedness as a component of the self-concept, referring to a relational cognitive schema that reflects individuals’ subjective feelings of closeness within their social relationships. Social crowding environments lead individuals to experience greater self-other overlap (Tan et al., 2015), increasing their perception of commonality with others. This, in turn, fosters a sense of identity unity and generates stronger interpersonal intimacy (Aron et al., 1992), as well as an enhanced sense of social connectedness characterized by the degree of self-other overlap.

The enhanced sense of social connectedness induced by social crowding further increases individuals’ willingness to refute rumors. Since engaging in rumor refutation provides informational support to others and benefits others and society, it is an altruistic form of prosocial behavior. Previous research has shown that stronger social connectedness promotes individuals’ helping behaviors. He and Wei (2009) found that individuals with stronger social connectedness to others and groups are more inclined to contribute to the group through information sharing, emotional support, and interpersonal cooperation—that is, online social relationships facilitate individuals’ willingness to help others online. Similarly, J. Watson et al. (2017) found that individuals are more likely to receive emotional and informational support from closer online contacts. Scholars have also introduced social connectedness-related variables into research on rumor refutation. Specifically, Tang et al. (2022) found that individuals with close social ties to other members of social media platforms demonstrated a stronger willingness to combat COVID-19-related rumors. Therefore, from an altruistic perspective, when the social connectedness is activated by physical proximity, individuals may perceive the online community as an extension of their immediate social circle, thereby motivating them to engage in digital prosocial behaviors as a means of safeguarding their connected peers. Accordingly, the following hypothesis is proposed:

**H3.**  *Social connectedness mediates the relationship between social crowding and individuals’ willingness to refute rumors.*

### 2.5. The Moderating Role of Self-Construal

Self-construal is a cognitive structure that reflects how individuals perceive the relationship between the self and others (Markus & Kitayama, 1991), comprising two types: independent self-construal and interdependent self-construal. Individuals with an independent self-construal detach themselves from social context, prioritize maintaining their uniqueness, resist being controlled, and tend to pursue independence, express themselves, and safeguard personal interests. In contrast, individuals with an interdependent self-construal view themselves as part of interpersonal relationships or social groups, are more inclined to establish social connectedness with others, exercise self-restraint, and may even sacrifice their own interests, exhibiting greater cooperative behaviors (Liu & Li, 2009; Utz, 2004). Given that rumor refutation is an interactive behavior directed toward others within social networks, this study proposes that self-construal, as an individual cognitive factor, moderates the effects of social crowding on impression management and social connectedness.

On the one hand, independent self-construal positively moderates the effect of social crowding on impression management. Individuals with an independent self-construal tend to engage in self-presentation, presenting themselves as skilled and capable through self-expression (Lalwani & Shavitt, 2009). Therefore, when social crowding leads to high self-other overlap, individuals with a high independent self-construal may have a stronger desire to maintain their uniqueness to differentiate themselves from others in the environment, resulting in higher motivation for impression management and thus a greater inclination to shape their distinctive self-image through rumor refutation.

On the other hand, interdependent self-construal positively moderates the effect of social crowding on social connectedness. Individuals with a high interdependent self-construal perceive themselves as part of the surrounding group and are more sensitive to social relationship cues. Consequently, in social crowding environments, they are more likely to experience interpersonal intimacy, perceive higher levels of social connectedness, and engage in rumor refutation behaviors that benefit others. In summary, the following hypotheses are proposed:

**H4.**  *Independent self-construal positively moderates the positive relationship between social crowding and impression management.*

**H5.**  *Interdependent self-construal positively moderates the positive relationship between social crowding and social connectedness.*

Based on the above hypotheses, this study further proposes a moderated mediation effect hypothesis, that is, the indirect effects of social crowding on the willingness to refute rumors through impression management and social connectedness are respectively moderated by independent self-construal and interdependent self-construal.

**H6.**  *Independent self-construal positively moderates the indirect effect of social crowding on the willingness to refute rumors through impression management. Specifically, the higher the level of independent self-construal, the stronger this indirect effect.*

**H7.**  *Interdependent self-construal positively moderates the indirect effect of social crowding on the willingness to refute rumors through social connectedness. Specifically, the higher the level of interdependent self-construal, the stronger this indirect effect.*

### 2.6. Overview of Studies

We tested the proposed hypotheses with one field survey and two scenario-based experiments to provide complementary and progressively rigorous evidence. Study 1 employed a questionnaire survey conducted in a typical social crowding setting—the school canteen—to preliminarily examine the relationship between social crowding and willingness to refute rumors (H1). Study 2 was an online experiment that manipulated social crowding using textual scenario materials (Sng et al., 2017) to further establish the causal relationship between the two variables (H1). Study 3 adopted a different manipulation method, using silhouette images (O’Guinn et al., 2015) to induce social crowding, and tested the full model, investigating the underlying mechanisms and boundary conditions of the effect of social crowding on willingness to refute rumors (H2–H7).

## 3. Study 1

Study 1 aims to provide an initial test of the relationship between social crowding and individuals’ willingness to refute rumors through a field survey, specifically testing H1, which posits that higher social crowding increases willingness to refute rumors.

### 3.1. Method

#### 3.1.1. Participants and Design

Study 1 employed a field survey design. One hundred questionnaires were distributed through an on-campus forum at a university. After excluding 14 questionnaires from participants who had never been exposed to rumor-refuting information or who failed the attention check, the final sample comprises 86 participants (51.16% female; *M_age_* = 20.77, *SD_age_* = 2.98).

#### 3.1.2. Procedure and Materials

To ensure that the survey fit the cafeteria context, participants first reported which cafeteria they were currently in and evaluated their satisfaction with the hygiene and food of that cafeteria. They then rated their perceived level of crowding in the current cafeteria environment (1 = very uncrowded, 7 = very crowded).

Next, participants were instructed to imagine that, at that moment, they received a notification on their mobile phone from an online rumor-refuting platform that a rumor currently under heated discussion had been debunked. Based on their current feelings, they then rated their willingness to refute the rumor. Willingness to refute rumors was measured with four items adapted from Lim (2017) and Sun et al. (2022): “I would share this rumor-refuting information on social media such as Weibo or WeChat,” “I would send or reply to messages to inform others and correct false rumors related to the event,” “I would leave comments to rebut or correct this rumor,” and “When I find that someone continues to spread this rumor, I would try to stop him or her” (1 = strongly disagree, 7 = strongly agree). In this study, the scale showed acceptable internal consistency (*α* = 0.77). Finally, demographic information, including age and gender, was collected.

### 3.2. Results

Hierarchical regression analysis is conducted to test the hypothesis. The results show that, after controlling demographic variables, social crowding has a significant positive effect on individuals’ willingness to refute rumors (*B* = 0.24, *SE* = 0.09, *p* < 0.01). Thus, H1 is supported.

### 3.3. Discussion

By measuring perceived social crowding in a cafeteria setting and individuals’ online willingness to refute rumors, Study 1 shows that social crowding positively affects willingness to refute rumors. In other words, the higher the level of social crowding, the stronger the willingness to refute rumors. These findings provide evidence with external validity for H1.

## 4. Study 2

Study 2 builds on the main effect established in Study 1 to further examine the effect of social crowding on individuals’ willingness to refute rumors through a scenario experiment. Specifically, this study manipulates social crowding to test whether individuals in a high social crowding condition show a stronger willingness to refute rumors than those in a low social crowding condition (H1).

### 4.1. Method

#### 4.1.1. Participants and Design

We conducted a one-factor, two-level (social crowding: high vs. low) between-subjects experiment to test H1. A total of 90 participants were recruited via Credamo, a professional online data collection platform. After excluding 11 participants who had never been exposed to rumor-refuting information or who failed the attention check, the final sample consisted of 79 participants (67.50% female; *M_age_* = 25.34, *SD_age_* = 8.03).

#### 4.1.2. Procedure and Materials

This study manipulated social crowding using scenario-based textual materials adapted from Sng et al. (2017). Participants were randomly assigned to either a high social crowding condition (*N* = 41) or a low social crowding condition (*N* = 38).

In high social-crowding conditions, participants read the following scenario: On a sunny Saturday, Xiaozhang and several friends plan to relax in a park. When they arrive, they are surprised to find that the park is crowded with people. Xiaoli and her friends seek a place to engage in activities, but they still cannot secure sufficient space. They try to play badminton in a narrow area or find an unoccupied swing, but there are simply too many people, and they are unable to find one. Xiaozhang then says that he feels very cramped and oppressed, that there are too many people and no open space, and that the place now looks like any other place, with people everywhere.

In low social-crowding conditions, participants read the following scenario: On a sunny Saturday, Xiaozhang and several friends plan to relax in a park. Upon arrival, they are pleased to find that there are few people in the park. Because the space is large, Xiaoli and her friends can move around freely. They find an open area to play badminton and easily locate an unoccupied swing to sit and rest. Overall, there are very few people, and they can go wherever they want. Xiaozhang then says that he feels very relaxed and at ease, that there are few people and a great deal of open space, and that the place now looks like any other place, with open space everywhere.

After the manipulation, participants completed a social crowding item for the manipulation check (1 = very uncrowded, 7 = very crowded). The scale showed acceptable internal consistency (*α* = 0.82). Participants then completed the willingness to refute rumors scale used in Study 1, which also showed acceptable internal consistency in this study (*α* = 0.72).

In addition, to control for participants’ emotional states during the experiment, we adopted the emotional state scale developed by D. Watson et al. (1988). Specifically, participants reported their level of positive emotion (1 = very negative, 7 = very positive), happiness (1 = very unhappy, 7 = very happy), and anxiety (1 = very calm, 7 = very anxious). Finally, participants provided demographic information, including age and gender.

### 4.2. Results

#### 4.2.1. Manipulation Checks

We first conduct independent-samples *t* tests on the demographic variables and find that the two groups do not differ significantly in gender, age, or education level. We then conduct an independent-samples *t*-test on perceived social crowding. The results show that participants in the high social crowding condition report a significantly higher level of social crowding (*M* = 6.55, *SD* = 0.55) than those in the low social crowding condition (*M* = 1.28, *SD* = 0.46, *t*(77) = 46.17, *p* < 0.001). Indicating that the manipulation of social crowding is successful.

In addition, participants in the high social crowding condition report significantly lower positive emotion than those in the low social crowding condition (*t*(77) = −19.67, *p* < 0.001). They also report significantly lower happiness (*t*(77) = −23.08, *p* < 0.001), and significantly higher anxiety (*t*(77) = 22.93, *p* < 0.001).

#### 4.2.2. The Impact of Social Crowding on Willingness to Refute Rumors

An independent-samples *t*-test on willingness to refute rumors shows that participants in the high social crowding condition report significantly stronger willingness to refute rumors (*M* = 4.99, *SD* = 0.86) than those in the low social crowding condition (*M* = 4.31, *SD* = 0.63, *t*(77) = 3.96, *p* < 0.001, Cohen’s *d* = 0.76). This finding indicates that social crowding has a significant positive effect on individuals’ willingness to refute rumors. Thus, H1 is supported again.

### 4.3. Discussion

The findings of Study 2 are consistent with those of Study 1 by showing that social crowding increases individuals’ willingness to refute rumors. By manipulating social crowding through scenario materials, this study provides another replication of H1, demonstrates the robustness of the effect, and offers strong internal validity through an experimental design.

At the same time, the results show that the social crowding manipulation used in this study produces significant differences in participants’ emotional states across conditions. Under such circumstances, it is difficult to rule out the possibility that emotion also plays a role in the effect of social crowding on willingness to refute rumors. Therefore, the subsequent study adopts a social crowding manipulation that minimizes its impact on emotion as much as possible, tests whether participants’ emotions still differ significantly across conditions, and includes emotion as a control variable in the subsequent analyses.

## 5. Study 3

Study 3 builds on the main effect established in Studies 1 and 2 to further examine the underlying mechanism and boundary conditions through which social crowding affects individuals’ willingness to refute rumors. Specifically, this study tests the parallel mediating roles of impression management and social connectedness in the relationship between social crowding and willingness to refute rumors (H2, H3), as well as the moderating roles of independent self-construal and interdependent self-construal, together with the corresponding moderated mediation effects, in this process (H4–H7), thereby providing a full test of the overall research model.

### 5.1. Method

#### 5.1.1. Participants and Design

We conducted a one-factor, two-level (social crowding: high vs. low) between-subjects experiment. A total of 160 participants were recruited via Credamo. After excluding 11 participants who had never been exposed to rumor-refuting information or who failed the attention check, the final sample consisted of 146 participants (64.38% female; *M_age_* = 27.84, *SD_age_* = 7.36).

#### 5.1.2. Procedure and Materials

**Figure 1:**
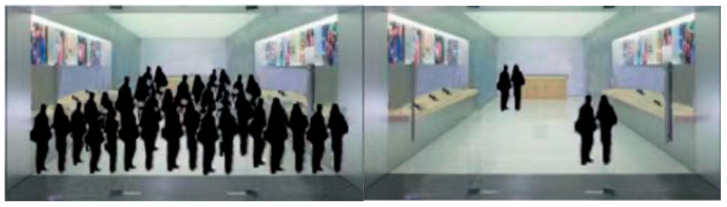
Pictures of the social crowding group and the non-social crowding group.

This study manipulated social crowding using image materials adapted from Huang et al. (2018), Maeng et al. (2013), and O’Guinn et al. (2015). Prior research suggested that manipulating social crowding with real-scene photographs from natural settings can readily produce significant emotional differences between conditions. Therefore, to minimize emotional interference, this study used silhouette images in a non-naturalistic setting to manipulate social crowding. Participants were randomly assigned to either a high social crowding condition (*N* = 71) or a low social crowding condition (*N* = 75). In the high social crowding conditions, participants were told that they were viewing a real-scene picture containing 32 silhouette figures. In the low social crowding conditions, participants were told that they were viewing a real-scene picture containing 4 silhouette figures (see Figure 1).

After the manipulation, participants completed the social crowding item used in Study 2 as a manipulation check; the scale showed acceptable internal consistency (*α* = 0.83). Participants then completed the willingness to refute rumors scale used in Study 1, and the scale showed acceptable internal consistency in this study (*α* = 0.79). Impression management was measured with the scale developed by Kristofferson et al. (2014), which contains four items: “I want to present myself to others in a positive way,” “I do not care how others see me,” “I want to leave others with a positive impression,” and “I want to make myself look good to others”. Social connectedness was measured with the scale developed by Lee and Robbins (1995), which contains seven items: “I feel closely connected to the world around me,” “I feel a sense of belonging even in the presence of people I do not know,” “I feel close to people in the world, I feel a sense of solidarity with others,” “I feel related to anyone in the world,” “I find myself losing all sense of connection with society,” and “I feel a sense of belonging to all groups”. Self-construal was measured with the scale developed by Wang et al. (2015), which contains seven items. Independent self-construal comprises four items: “having personal characteristics that are independent of others is important to me,” “expressing my own ideas in group discussions,” “having my own views on most things and knowing what I like and dislike,” and “liking my unique qualities”. Interdependent self-construal comprises three items: “my sense of happiness depends on the happiness of the group I belong to,” “I can sacrifice my own interests for the benefit of the group,” and “in group discussions, I pay attention to my wording so as not to offend others”. In addition to the demographic and emotional variables collected in Study 2, participants also reported the extent to which they adapt to crowded environments in daily life (1 = very maladapted, 7 = very adapted; Evans et al. (2000)).

### 5.2. Results

#### 5.2.1. Manipulation Checks

We first conducted an independent-samples *t*-test on perceived social crowding. The results show that participants in the high social crowding conditions report a significantly higher level of social crowding (*M* = 6.31, *SD* = 0.69) than those in the low social crowding condition (*M* = 1.46, *SD* = 0.55), *t*(144) = 47.00, *p* < 0.001, indicating that the manipulation of social crowding is successful. In addition, participants in the high social crowding conditions report significantly lower positive emotion than those in the low social crowding conditions (*t*(144) = −11.40, *p* < 0.001), significantly lower happiness (*t*(144) = −11.23, *p* < 0.001), and significantly higher anxiety (*t*(144) = 13.95, *p* < 0.001). The two groups do not differ significantly in their adaptation to crowded environments (*t*(144) = −0.59, *p* = 0.56).

#### 5.2.2. The Impact of Social Crowding on Willingness to Refute Rumors

We code the social crowding condition as a binary variable, with high social crowding coded as 1 and low social crowding coded as 0. A hierarchical regression analysis shows that, after controlling demographic variables, emotional variables, and adaptation to crowded environments, social crowding has a significant positive effect on willingness to refute rumors (*B* = 0.96, *SE* = 0.32, *p* < 0.01). This result indicates that social crowding significantly increases individuals’ willingness to refute rumors. Thus, H1 is supported again.

#### 5.2.3. The Parallel Mediating Effects of Impression Management and Social Connectedness

We use PROCESS Model 4 to test parallel mediating effects, with impression management and social connectedness entered simultaneously as mediators. The results show that, after controlling all variables, social crowding has a significant positive effect on impression management (*B* = 1.40, *SE* = 0.23, *p* < 0.001) and on social connectedness (*B* = 0.79, *SE* = 0.32, *p* = 0.01). In addition, impression management has a significant positive effect on willingness to refute rumors (*B* = 0.34, *SE* = 0.11, *p* < 0.01), and social connectedness also has a significant positive effect on willingness to refute rumors (*B* = 0.34, *SE* = 0.08, *p* < 0.001).

When impression management and social connectedness are entered simultaneously, the direct effect of social crowding on willingness to refute rumors is no longer significant (*B* = 0.22, *SE* = 0.32, *p* = 0.50). The bootstrap results further show that the indirect effect of social crowding on willingness to refute rumors through impression management is significant (indirect effect = 0.47; *SE* = 0.21; 95% CI [0.11, 0.94]). The indirect effect through social connectedness is also significant, with an indirect effect of 0.27 (*SE* = 0.14; 95% CI [0.02, 0.55]). These results indicate that impression management and social connectedness play parallel mediating roles in the relationship between social crowding and willingness to refute rumors. Thus, H2 and H3 are supported.

#### 5.2.4. The Moderating Effects of Self-Construal

We use PROCESS Model 7 to test the moderating roles of independent self-construal in the relationship between social crowding and impression management, and of interdependent self-construal in the relationship between social crowding and social connectedness.

**Figure 2:**
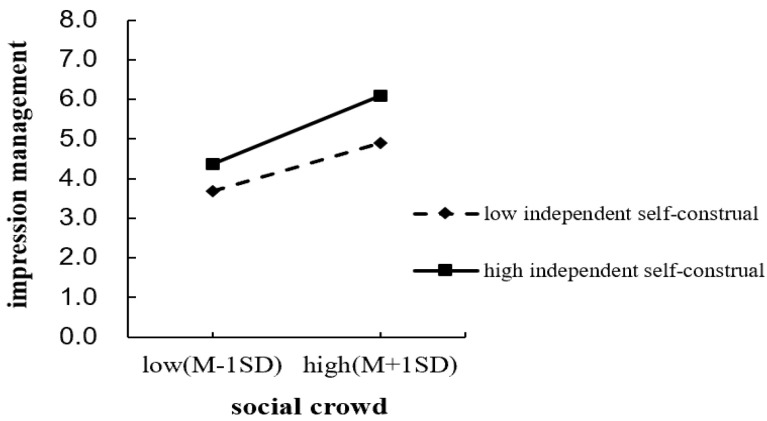
Moderating effect of independent self-construal.

For independent self-construal, the results show that, after controlling for all variables, the interaction between social crowding and independent self-construal does not significantly affect impression management (*B* = 0.17, *SE* = 0.10, *p* = 0.08). Simple slope analyses (see Figure 2) show that social crowding significantly increases impression management both among individuals with low independent self-construal (*B* = 1.21, *SE* = 0.24, 95% CI [0.74, 1.68], *p* < 0.001) and among individuals with high independent self-construal (*B* = 1.74, *SE* = 0.24, 95% CI [1.27, 2.21], *p* < 0.001). Therefore, independent self-construal does not play a significant moderating role in the relationship between social crowding and impression management.

**Figure 3:**
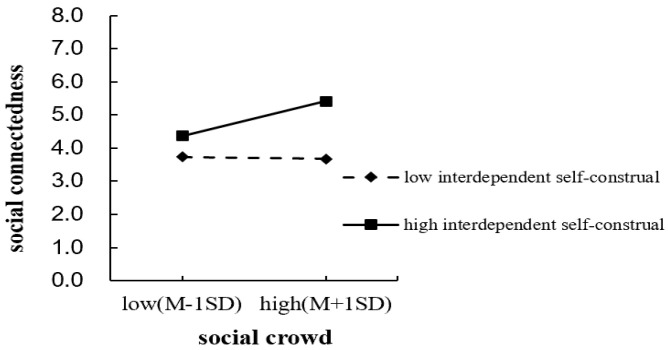
Moderating effect of interdependent self-construal.

For interdependent self-construal, the results show that, after controlling all variables, the interaction between social crowding and interdependent self-construal significantly affects social connectedness (*B* = 0.43, *SE* = 0.14, *p* < 0.01). Simple slope analyses (see Figure 3) show that, for individuals with high interdependent self-construal, social crowding has a significant positive effect on social connectedness (*B* = 1.03, *SE* = 0.32, 95% CI [0.40, 1.68], *p* < 0.01). In contrast, for individuals with low interdependent self-construal, the effect of social crowding on social connectedness is not significant (*B* = −0.07, *SE* = 0.33, 95% CI [−0.72, 0.59], *p* = 0.84). These results indicate that interdependent self-construal positively moderates the relationship between social crowding and social connectedness. Thus, H4 is not supported, whereas H5 is supported.

#### 5.2.5. The Moderated Mediation Effects

We further test the moderate mediation effects. For independent self-construal, when individuals’ level of independent self-construal is low, the indirect effect of social crowding on willingness to refute rumors through impression management is significant (*B* = 0.57, *SE* = 0.26, 95% CI [0.10, 1.09]). When individuals’ level of independent self-construal is high, this indirect effect is also significant (*B* = 0.81, *SE* = 0.25, 95% CI [0.33, 1.33]). The difference between the two effects is not significant (Δ*B* = 0.25, *SE* = 0.19, 95% CI [−0.12, 0.65]). This result indicates that independent self-construal does not significantly moderate the indirect effect of social crowding on willingness to refute rumors through impression management. Therefore, H6 is not supported.

**Figure 4:**
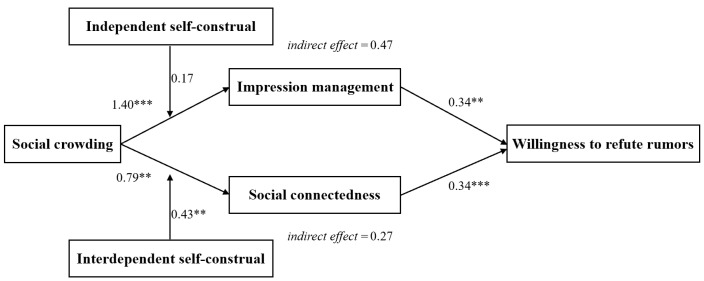
Results of multi-level path analysis. Note: The report presents unstandardized coefficients. For brevity, the path coefficients of control variables have been omitted. ** *p* < 0.01, *** *p* < 0.001.

For interdependent self-construal, when individuals’ level of interdependent self-construal is low, the indirect effect of social crowding on willingness to refute rumors through social connectedness is not significant (*B* = −0.03, *SE* = 0.15, 95% CI [−0.33, 0.26]). When individuals’ level of interdependent self-construal is high, this indirect effect is significant (*B* = 0.42, *SE* = 0.14, 95% CI [0.16, 0.71]). The difference between the two effects is significant (Δ*B* = 0.45, *SE* = 0.16, 95% CI [0.15, 0.77]). Therefore, H7 is supported. The results of the multi-level path analysis are shown in Figure 4.

### 5.3. Discussion

Study 3 further verifies the mechanism and boundary conditions underlying the effect of social crowding on individuals’ willingness to refute rumors. The results show that impression management and social connectedness play parallel mediating roles in the relationship between social crowding and willingness to refute rumors, supporting H2 and H3. At the same time, interdependent self-construal positively moderates the effect of social crowding on social connectedness and further moderates the indirect effect of social crowding on willingness to refute rumors through social connectedness, supporting H5 and H7. In contrast, the moderating role of independent self-construal and its corresponding moderated mediation effect are not supported, and thus H4 and H6 are not supported. In addition, although the social crowding manipulation used in this study affects participants’ emotions, the relevant conclusions remain unchanged after emotion is included as a control variable.

## 6. General Discussion

Grounded in the moral judgment framework of prosocial behavior, this study systematically investigates the influence of social crowding on individuals’ willingness to refute rumors, along with its underlying mechanisms and boundary conditions. Study 1 collected data through a questionnaire survey in a typical social crowding scenario—the school canteen—and preliminarily confirmed the positive relationship between social crowding and willingness to refute rumors. Study 2 employed an online scenario-based experiment using textual manipulations of social crowding, further establishing the causal relationship between social crowding and willingness to refute rumors. Study 3 also adopted a scenario-based experimental design using pictorial manipulations of social crowding and further confirmed the parallel mediating roles of impression management and social connectedness in the relationship between social crowding and willingness to refute rumors, as well as the positive moderating role of interdependent self-construal in the relationship between social crowding and social connectedness.

Most of the hypotheses proposed in this study were empirically supported. However, the results of Study 3 revealed that independent self-construal did not moderate the relationship between social crowding and impression management. One potential theoretical explanation for this non-significant result is the possible distinction between self-focused and other-focused impression management strategies (Kacmar et al., 1992). Self-focused impression management refers to individuals managing their self-image by showcasing and enhancing themselves, which is more directly driven by an independent self-construal that emphasizes personal uniqueness. In contrast, other-focused impression management involves individuals managing their self-image by aligning with others’ thoughts and endorsing others’ perspectives, which entails greater interpersonal interaction and relationship management. Independent self-construal was expected to enhance self-focused impression management. However, in social crowding environments, due to physical proximity to others, individuals may more naturally adopt other-focused rather than self-focused impression management strategies. Consequently, even individuals with high independent self-construal may refrain from demonstrating uniqueness and engaging in impression management under crowded conditions, instead choosing to align with others. This may render the moderating effect of independent self-construal non-significant. Previous research has also suggested that independent self-construal weakens individuals’ motivation to engage in other-oriented helping behaviors (McClintock & Allison, 1989). However, as the current research did not empirically distinguish these two specific sub-dimensions of impression management, this interpretation remains a speculative possibility that future research could examine by measuring or manipulating these sub-dimensions.

### 6.1. Theoretical Implications

First, this study extends research on online rumor refutation by establishing a theoretical bridge between physical environmental states and digital prosocial actions. Although internet users represent an important force in rumor refutation, few studies have explored rumor governance strategies from the perspective of motivating individuals to engage in rumor refutation. Existing research has largely focused on the influence of rumor refutation message characteristics, such as forwarding volume (Zhao et al., 2016), while overlooking the role of social environmental factors. Therefore, by taking social crowding—a common experience in daily life—as the independent variable, this study contributes to addressing a research gap regarding the relationship between social crowding and willingness to refute rumors, thereby enriching research on rumors and rumor refutation from an environmental antecedents perspective. Furthermore, this study considers individual rumor refutation behavior from a prosocial behavior perspective and, drawing on the moral judgment framework, comprehensively accounts for the psychological motivations underlying individuals’ engagement in rumor refutation from both egoistic and altruistic perspectives. The findings provide a theoretical framework for future research exploring other factors influencing individuals’ willingness to refute rumors, while also contributing to a deeper understanding of online prosocial behavior and enriching theoretical outcomes in social media research.

Second, this study broadens the scope of behavioral outcomes associated with social crowding. Previous research has paid limited attention to the potential positive effects of social crowding and has mostly focused on consumer behavior (Ha & Lee, 2016). However, a key characteristic of social crowding is interpersonal interaction within a specific environment, where the influence of human factors becomes more prominent among various situational factors. Accordingly, by proposing the influence mechanism between social crowding and willingness to refute rumors through both egoistic and altruistic pathways, we revealed the internal psychological mechanisms through which social crowding influences willingness to refute rumors, while enriching research on impression management and social connectedness. More importantly, by demonstrating that the psychological states induced by physical crowding can spill over into the digital realm, this study shifts the focus of crowding research from offline consumer outcomes (Harrell et al., 1980) to online behavioral consequences. Establishing a bridge between physical environmental states and digital actions reflects the increasing permeability of physical and digital boundaries (Humphreys, 2013). By empirically demonstrating this crossover effect, we show that the psychological effects of the self-other overlap activated by physical proximity can transfer to the online context, thereby expanding the theoretical reach of environmental psychology into the digital age.

Furthermore, this study addresses an important theoretical tension within crowding research. Classical environmental psychology has long associated crowding with withdrawal, loss of control, and the diffusion of responsibility (Harrell et al., 1980; Hui & Bateson, 1990). Unlike previous studies that have primarily focused on these negative consequences, this study shifts the theoretical focus to the increased self-other overlap induced by physical proximity. The present findings suggest that crowding can facilitate rather than suppress online prosocial behavior. One possible explanation for this apparent reversal is that online rumor refutation is a low-cost, socially visible action that does not require additional physical interaction with the surrounding crowd. In such a digital context, the self-other overlap triggered by physical proximity may activate positive interpersonal tendencies rather than avoidance, thereby offering a more balanced perspective on the consequences of social crowding.

Finally, to identify the boundary conditions of the effect of social crowding on willingness to refute rumors, this study adopted a self-other relationship perspective and validated the moderating role of interdependent self-construal—a social relationship cognition—in the relationship between social crowding and social connectedness. These findings enrich the moderating mechanisms underlying the effect of social crowding on willingness to refute rumors and provide explanatory insights into how social crowding differentially influences individuals with varying cognitive characteristics.

### 6.2. Practical Implications

This study holds significant practical implications for encouraging public engagement in internet rumor refutation governance. First, this study demonstrates that social crowding serves as a situational catalyst that can enhance individuals’ willingness to refute rumors. This suggests that everyday crowded environments, such as subway stations, cafeterias, or commercial districts during peak hours, may provide opportune settings or moments for delivering rumor-refutation appeals. For instance, relevant institutions could push non-intrusive promotional messages during periods of higher social crowding (e.g., holidays or rush hours) or display refutation posters in crowded transit hubs. Furthermore, digital platforms may benefit from exploring interface designs that subtly evoke a sense of social density, such as incorporating dense crowd imagery or displaying the number of users currently viewing or engaging with the refutation content.

Second, this study reveals that rumor refutation is driven by two distinct psychological pathways: an egoistic route through impression management and an altruistic route through social connectedness. This dual-pathway finding suggests that practitioners can encourage refutation by either highlighting the collective benefits to stimulate social connectedness (e.g., emphasizing the collective benefits of sharing refutation information) or by providing opportunities for users to enhance their self-image through impression management (e.g., displaying refutation badges or notifying friends of the user’s proactive sharing).

### 6.3. Limitations and Future Research

This study has several limitations, which also suggest directions for future research. First, the sample sizes in the present studies were relatively modest, and future research should recruit larger and more diverse samples to ensure the robustness and generalizability of the findings. Moreover, as the present research relied primarily on convenience and online panel samples, future research could therefore strengthen external validity by adopting more representative sampling strategies, such as stratified sampling approaches that systematically account for key demographic factors, including geographical region, age, and educational background.

Regarding research methodology, all three studies were conducted through online questionnaires, which may weaken the ecological validity of the findings. Future research should recruit participants offline for laboratory experiments, and especially conduct field studies in more ubiquitous and high-intensity naturalistic settings, such as subways during the morning rush hour or shopping malls during major holidays, to collect data with better external validity. Regarding the manipulation of social crowding, although textual descriptions and silhouette images are validated approaches in the previous literature (Sng et al., 2017; Huang et al., 2018), relying on these symbolic stimuli may be too abstract to elicit the same level of visceral psychological stress as real-world crowding. Therefore, future studies could employ real-environment audio recordings of real crowded environments (e.g., conversations or white noise in crowded settings) or use virtual reality (VR) devices to create immersive experiences (Sng et al., 2017). Alternatively, researchers could manipulate social crowding through actual laboratory crowding by varying the number of participants who enter the laboratory simultaneously to complete questionnaires (Maeng et al., 2013). Notably, the manipulation may inadvertently alter participants’ emotional states. Although we addressed this by statistically controlling for emotional variables and utilizing silhouette images to minimize potential interference, future research could employ orthogonal designs to experimentally disentangle the pure sensation of crowding from affect (e.g., comparing crowded but enjoyable festive events with crowded and stressful commuting scenarios). Additionally, incorporating physiological measures, such as skin conductance or heart rate variability, would provide objective indicators to further isolate the unique psychological impact of crowding.

With respect to the measurement of willingness to refute rumors, the current research relied on self-reported intentions in response to hypothetical scenarios rather than actual rumor-refuting behavior, which may fail to capture the spontaneous nature of real-world responses. Future research could be improved in the following aspects. First, providing more specific scenarios to increase realism, such as presenting participants with a concrete, real-world rumor and corresponding refutation information, and then assessing their willingness to forward the refutation; second, incorporating a broader range of rumor refutation behaviors into the measurement, such as active evidence-seeking, posting clarification messages, or reporting rumors; third, future studies can shift the focus from self-reported intentions to actual behavioral indicators. Objective secondary behavioral data can be collected through technical means such as web crawlers and big data mining to avoid the limitations associated with subjective self-report measures.

Furthermore, the two mediating variables (impression management and social connectedness) and the dependent variable (willingness to refute rumors) were measured at the same time point following the social crowding manipulation. While this is a common practice in psychological experiments, it limits our ability to definitively establish a temporal causal sequence. Future research should consider experimentally manipulating the mediators (e.g., using priming tasks to satisfy the need for impression management) to provide more robust evidence for the causal chain. Similarly, the moderating variable in this study, self-construal, is a malleable cognitive characteristic that can be influenced by external stimuli. Accordingly, future experimental studies can adopt priming methods to temporarily activate different types of self-construal, such as the pronoun-circling priming task (Brewer & Gardner, 1996), to further improve the reliability and robustness of the research findings.

For example, factors associated with moral motivation, such as personal norms, can be further incorporated into the analysis of psychological determinants underlying individuals’ engagement in Internet rumor refutation behaviors. Furthermore, although this study has examined the moderating role of self-construal, future research could investigate whether the effect of social crowding on rumor refutation intention is also moderated by other situational factors or individual difference variables.

Meanwhile, group attributes may also serve as a critical moderating variable. For instance, differences may exist between the canteen setting (ingroup) and subway setting (outgroup) adopted in Study 1, suggesting that future field studies can be conducted in diverse contextual settings to examine the moderating role of ingroup bias on the mediating path of social connectedness (Sturmer et al., 2006). Additionally, the distinct characteristics of different social media platforms, such as public-oriented platforms like Weibo versus private social platforms like WeChat, may shape the extent to which rumor refutation behaviors satisfy individuals’ altruistic motivation and impression management motivation. This cross-platform difference represents a promising avenue worthy of further in-depth investigation.

Notably, this study does not assume that the relationship between Social Crowding and Rumor Refutation intention is consistently linear. Prior research has demonstrated that when the level of social crowding becomes excessively high, and its stimulation intensity exceeds the optimal threshold that individuals can tolerate, it may transform into a negative affective experience. Such excessive crowding tends to trigger individuals’ tendency to avoid interpersonal communication and resist interactions with others (Baum & Koman, 1976), exhibit aversion to anthropomorphic products (Puzakova & Kwak, 2017), while conversely becoming more inclined to share information with online contacts (Consiglio et al., 2018) and pay closer attention to mobile phone advertisements (Andrews et al., 2015).

Accordingly, a quadratic relationship may exist between social crowding and rumor refutation intention. Specifically, when social crowding surpasses a certain critical threshold, the positive effect of social crowding on individuals’ rumor refutation intention will reverse; that is, as the degree of crowding continues to rise beyond this point, individuals’ willingness to engage in Internet rumor refutation may gradually decline instead. Therefore, subsequent research can adopt methods such as setting multiple groups of social crowding scenarios with distinct gradient levels to explore the boundary conditions of this nonlinear effect.

## 7. Conclusions

This study explores the impact of social crowding on individuals’ willingness to refute rumors. Across three studies, we found that social crowding positively influences willingness to refute rumors, and this effect is driven by two parallel pathways: an egoistic route via the mediator of impression management and an altruistic route via the mediator of social connectedness. Additionally, interdependent self-construal positively moderates the relationship between social crowding and social connectedness. Based on these findings, governments and social media platforms could strategically leverage social crowding contexts and peak times, and design rumor-refutation campaigns that activate individuals’ egoistic and altruistic motivations to encourage public participation in rumor refutation.

## Data Availability

The data presented in this study are available on request from the corresponding author due to privacy and ethical restrictions.
